# Enhancing the role of mass media in the translation of evidence from health policy and systems research in Nigeria

**DOI:** 10.1186/s12961-024-01267-8

**Published:** 2025-01-30

**Authors:** Prince Agwu, Chinyere Mbachu, Obinna Onwujekwe

**Affiliations:** 1 Health Policy Research Group, Department of Pharmacology and Therapeutics, College of Medicine, University of Nigeria Enugu Campus, Enugu, Nigeria; 2 Education and Society, University of Dundee, Dundee, UK; 3 Department of Community Medicine, University of Nigeria, Enugu, Nigeria; 4 Department of Health Administration and Management, College of Medicine, University of Nigeria Enugu Campus, Enugu, Nigeria; 5 Department of Social Work, University of Nigeria, Enugu, Nigeria

**Keywords:** Evidence-based policy-making, Evidence translation, Health policy, Mass media, Media, Social media, Policy actions

## Abstract

**Background:**

There are massive gaps in communication between health researchers and policy-makers in Nigeria, which constrains the use of research evidence for policy-making. Mass media can help in bridging the gaps, especially since the media has the reach and a reputation for presenting information in ways that elicit actions from the public and policy-makers.

**Objective:**

There is a small body of emerging literature from Nigeria and sub-Saharan Africa, evidencing the usefulness of the media to encourage evidence translation in the health sector; and even evidence translation theories are light on dissemination. This paper adds to knowledge on how academia and media can be linked for effective dissemination of evidence for policy impact.

**Method:**

Data were sourced from group discussions in a communications workshop with 27 participants comprising researchers in health systems and policy and media professionals with several years of experience.

**Results:**

It was found that health research evidence conducted using quality procedures and published in quality academic journals barely make it to public and policy-making conversations because of the restrictiveness that characterizes academic outputs in traditional academic dissemination outlets. On the basis of the cultivation theory, the media was found instrumental in feedback of research results to communities, securing policy-makers’ reactions and stimulating policy actions.

**Conclusions:**

In line with message system analysis, researchers must be strategic in the use of mass media, and our results showed how it can be done. In all, media usage for evidence translation has enormous potential to strengthen the health system.

## Background

The mass media is an important dissemination avenue or knowledge broker for research outputs, including outputs from health-related research [1]. The contents from the mass media are often simple, connects well enough with a broad range of common people and non-technical audiences, and are considered authoritative in information dissemination [2, 3]. Despite the negative usage of media in stimulating and spreading propaganda, it has essentially contributed to advocacies, activism and generally enhancing the spread of topical concerns for government to take action, including those of health [4, 5].

It is common to see governments take actions on the basis of media reports, but similar actions based on academic publications – the hallmark of academia – are barely recorded [6]. Additionally, community people tend to be quick to respond to media reports on issues, usually for the sake of trust, relatability, and accessibility [6, 7]. Therefore, for academia to scale up impacts of its outputs, utilizing the comparative advantages of the media cannot be overstated. Such impact is at the heart of evidence translation, measured by the extent of reach and comprehensibility of research outcomes by the public and policy-makers towards taking actions [8, 9].

The media comprises print media (newspapers, bulletins, etc.,), electronic media (television, radio, etc.) and social media (Facebook, X, LinkedIn, etc.). Unlike print and electronic media functioning under extensive regulations, social media is not, offering rights to people to swiftly share their thoughts and feelings for public consumption [10, 11]. In communicating health research findings for policy actions, these various types of media clusters must be considered. For instance, electronic and print media were found useful by Niederdeppe et al. [12], as a means of mitigating the dangers of smoking in the USA. As news coverage of harmful use of tobacco on radio and print intensified, the enactment and implementation of tobacco product placement ordinances (TPPO) gained significant traction [12]. Similar findings exist in literature [6, 13, 14]. And in another study, social media communication of evidence could not reduce tobacco smoking but increased adherence to breastfeeding and exercise [15]. Social media was also useful in aligning offline reactions to the restrictive behavioural rules during the coronavirus 2019 (COVID-19) pandemic [11, 16]. So, both traditional and social media have comparative edges that are relevant to health policy and systems research (HPSR).

Nigeria is home to a broad range of mass media outlets. The National Broadcasting Corporation (NBC) in Nigeria reports that as of 2021, there were 625 broadcasting stations [17]. On media consumption, more than 70% and 35% of urban and rural households in Nigeria, respectively, said they own a television [18], and more than 77% of Nigerians across Nigeria’s six geopolitical zones listen to radio at least once a week [19]. Adding to these figures is the over 31 million active social media users in Nigeria as of January 2023, which is about 15% of the total population [20].

The massive number of media outlets in Nigeria shows the potential power of the mass media in terms of information spread, with the potential to benefit health research communication and evidence translation in the country. Aside from the focus of researchers on the media during the pandemic, there is a paucity of documented evidence on the vital contributions of the media to translating evidence in Health Policy and Systems Research (HPSR), especially in Nigeria. Documented experiences in getting research into policy and practice in health, tend to be light on the media, despite its extensive coverage and roles in politics, health and public behaviour [21, 22]. Our study moves ahead to identify and describe the usefulness of the media in bridging such gaps.

Therefore, to address the identified gap, the paper provides insights into the indispensable roles of the media in evidence translation, including how it should be used by researchers in HPSR in Nigeria. The research questions that generated the information for the paper were: (a) what gaps exist in the communication of research evidence that should be filled by the media?; (b) What are the practicable roles of the media in translating research evidence for policy and governance?; and (c) What should researchers know while using the media for evidence translation? Answers to these questions were derived from a 3-day bootcamp of diverse mass media professionals and researchers in Nigeria.

## Cultivation theory in health communication and evidence translation

Central to our work is the significance of media processes and effects, which has been extensively captured in the cultivation theory propounded by George Gerbner in the 1960s [23, 24]. The theory provides macro system insights into the effectiveness of the media on the public by creating meanings through messages [25]. These meanings could convey values, facts and expectations, in ways that can stimulate both immediate and sustained actions and reactions over time. This is what Gerbner referred to as ‘cultivation’ [26]. He contended that the meanings and notions generated by the media are usually shared because of the large public following the media enjoys, which is owing to their symbolic identity in societies as an avenue for information and the melting point of public opinions.

Cultivation theory has gained traction in health communication [27, 28]. However, it has been used more in clinical and public health conversations, as against evidence translation in health systems. Our research draws on insights from cultivation theory to explain how the media can help reconstruct meanings embedded in the research outputs of HPSR experts for both public and policy actions. It realizes the institutional advantage of the media with regard to messaging and public opinion and seeks to marry the institutional advantage of HPSR academia (generating credible research evidence) with that of the media.

Of course, an element of the cultivation theory discusses how public interpretation of the meanings of messages can be determined by the credibility of the institutions behind the messages [29]. Here, we see ‘two credibilities’ residing in the quality of research evidence/academia and in the quality of the status of media, which can be synergized for policy significance in the health sector. Unfortunately, this has not received much academic attention, which can account for the current gaps in translating evidence from HPSR into impactful health policies, practices and programmes for Nigeria.

## Methods

### Study context and sampling procedures

Our focus for the study was to gain deep insights into how the media can be effectively harnessed for the communication and use of health research evidence. To achieve this, we needed to rely on the experiences and feelings of media professionals and researchers towards the phenomenon of research communication and evidence use in health systems. Therefore, we used the phenomenological design in qualitative research to host a workshop (bootcamp) involving stakeholders from the Nigerian media industry and active health systems researchers. Among other research designs in qualitative research, phenomenological research design has the comparative advantage of tapping into real and perceived experiences of research participants in ways that suspend preconceived assumptions [30]. The residential bootcamp held over 3 days in April 2023 in Enugu, Nigeria, with 13 media professionals and 14 active health systems researchers was useful in interacting with the participants closely and iteratively about the phenomenon.

The criteria for selection of the participants included health systems-focused researchers who had no less than 3 years of experience in HPSR and had worked in at least two geopolitical zones in Nigeria, with any experience of engaging the media in research dissemination; media professionals with no less than 3 years of experience and can demonstrate the willingness to engage with researchers in conversations. We used purposive sampling to identify some researchers and media professionals within our network, who in line with snowballing sampling technique recommended other experts that were invited. As such, in all, 27 participants attended the bootcamp.

### Data collection

The rationale behind the workshop was to provide time for intense exploration of the phenomenon. First, we allowed the participants to make presentations on what they understand and know about the usefulness of media–research collaboration to advance the use of evidence in governance and policies in the health sector. Adopting some insights from a grounded theory approach [31], we teased more questions for discussions from the presentations, focusing on gaps, roles and strategies of using the media in evidence translation of research in health systems and policy. The questions were addressed in small groups of nine each, coordinated by the researchers who were assisted by research assistants. As expected of phenomenology, questions were designed to gain insights into lived and perceived experiences of the use of evidence on the basis of media-led research communication. We focused on ‘what has worked’, ‘what can(not) work’ and ‘what strategies can be taken’ to ensure health research evidence is translated into action by gaining the attention of policy-makers and the public. We also provided the opportunity to discuss how the media and researchers can achieve the concept of ‘two credibilities’.

The discussions in the workshop were recorded, and salient points documented in notes by our research assistants. Overall, data collection procedures were tailored to match the Consolidated Criteria for Reporting Qualitative Research (COREQ) framework [32]. It is important to mention that our research method was not without any challenges. First was to have the professionals make less use of jargon so that they could understand one another. We achieved this by kicking off with presentations, which provided the opportunity for questions and clarifications. Second was the management of data collected over 3 days, which we addressed by sticking with the core aims of the study captured in the research questions mentioned earlier.

### Data analysis

The discussions were first transcribed in English language. Transcripts were compared with the notes for consistency and correctness and analysed using thematic analysis. Thematic analysis is used to make sense of qualitative data by categorization of the descriptions of a phenomenon into themes and subthemes [34]. The themes were deductively generated from the research questions and inductively refined using the generated data. Upon agreement among the researchers to use themes that reflect (a) identifying the gaps in communication of research evidence, (b) the practical benefits of media in evidence communication of health research and (c) what researchers must know to effectively use the media health research communication, we designed the analysis template. The analysis template was designed in an Excel Sheet – the rows were used to enter the quotes and columns for participants. This meant that we could trace every quote to participants who owned them. Entering quotes into the respective themes in the analysis template was undertaken by two of the researchers and validated by the third. Discrepancies were resolved in a meeting. Using this iterative method helped us to maintain analytical rigour and the assurance that quotes accurately reflected the agreed themes.

### Ethical issues

Informed consents were given to the participants in the format of the bootcamp’s content and structure and they confirmed participation. Steps have been taken to ensure anonymity of participants. The study was approved by the institutional review board of the researcher’s institution.

## Results

The results are organized in four thematic areas that present: (a) the sociodemographic description of the participants, (b) gaps that exist in the communication of research evidence, (c) practicable roles of the media in translating research evidence and (d) what researchers should know while using the media for evidence translation.

### Sociodemographic attributes of participants

Relevant sociodemographic attributes to this research include the gender of participants, speciality, length of experience, practice focus by geographical coverage and media base and awareness of evidence translation. Table 1 shows the summary of the sociodemographic details of participants.

**Table 1:** Summary of sociodemographic description of the participants

	Frequency	Percentage
Researchers *N* = (14)		
Gender		
Male	7	50
Female	7	50
Speciality		
Health policy and systems research	14	100
Length of experience		
3–10 years	9	64.3
> 10	5	35.7
Research focus by location		
Southern or northern-Nigeria	8	57.1
Southern and northern-Nigeria	6	42.9
Aware of evidence translation	14	100
Media professionals *N* = 13		
Gender		
Male	7	53.9
Female	6	46.1
Media base		
Radio	3	23.0
Television	2	15.4
Print	6	46.2
Freelance	2	15.4
Length of experience		
3–10 years	4	30.8
> 10	9	69.2
Geographical coverage		
National	11	84.6
Regional	2	15.4
State	0	0
Aware of evidence translation	6	46.2

### Gaps in the communication of research evidence

Three kinds of gaps that the media can bridge in the communication of evidence from research were found. First, was the gap of understanding research findings by the public and policy-makers. Participants highlighted difficulties in understanding the contents of research publications owing to the use of specialized language, the distortions to readership owing to intext citations, and the length of research articles.

When I was a student, I hated reading academic articles because of the so many in-text citations. They make reading boring and very distorted. But I can spend time reading novels because they flow so well. It is the same as reading news articles because of the flow and simple words that are used [researcher, male]

A media professional added:

Research papers are too long, and a lot of people are not used to reading such kinds of articles. Don’t misunderstand me – Nigerians love news. I do not believe it when people say that Nigerians do not read, because I know the extent of coverage we get with the news we release. Just that they do not want it too long and filled with words that will either take them to the dictionary or to do another research to understand the word. So, find out how they want it and give it to them that way – simple and relatable [media professional, male, print]

The second gap we discovered was related to the feedback of research findings to interior locations. Some studies are conducted in hard-to-reach locations, but often, researchers do not return to those locations to feedback findings to their participants. Thus, members of such communities do not know the outcomes of the conducted research, and hence are deprived the chance of being aware of their problems, which they need to participate in governance by making more informed demands.

At times, as researchers, we go to interior places to carry out our studies. At times too we go to slums. We interview community people, health workers, community leaders in those areas, and we use their findings to develop scientific papers, engage policy actors, to improve situations. But I must say that it is not fair that we do not return to those areas to discuss the findings with them. This is where I know the media has to come in […] [researcher, male]

Lastly, participants mentioned gaps in identification and accessibility of quality research. Some media professionals said that because they were not trained to know the standards of excellently conducted studies, they could quote findings from poorly done studies. The researchers added that even those studies that were properly done could be published in closed access journals, and the media professionals might not know how or have the wherewithal to access them.

I recall how an editor of one of the dailies quoted a research on the number of persons that die per month from an ailment. I was taken aback because the figures were outrageous. I did not believe those figures. I had to look out for more credible sources and indeed, the editor was very wrong [media professional, female, print]

### Practicable roles of the media in translating research evidence

The findings showed that there were four potential major roles of the media in translating research evidence. They include advocacy, extensive coverage, verification and feedback. These roles all combine to elicit policy actions. On advocacy, instances were provided on how research outputs can form topical issues for media consumption which can attract attention of those affected by the problem to make more advocacies, with possible responses coming from those that can address the problem.

A team of researchers used our radio station to discuss their research on corruption in primary healthcare. People kept calling our phone lines for that programme confirming as true what the researchers were saying. It caused heat that led to the government sponsoring a different radio programme to appeal to the people and even gave out hotlines to call if they witness any corruption going on in any of the primary health centres [media professional, female, radio]

The above quote also connects with confirming the veracity of research findings. It also reflects feedback by providing communities an opportunity to know the results of research conducted with and for them. It is only when they are fed the results that they can participate in determining the veracity of research results, further intensifying the weight of the results. And this is made possible because of the extensive coverage of the media, which can get to those places the researcher might find too difficult to visit.

In one of the radio phone-in programmes we had, someone called from my village. My village is so far from the location of the radio station. I could not believe that the research issue we were discussing was also a serious issue in my village […] [researcher, male]

Again, on stimulation of policy action, one of the media professionals recounted an experience.

Some years ago, we were concerned about the multiple levies commercial transportation drivers are subjected to in Lagos. These levies are usually paid to touts. We had to use infographics to document how much the government loses to touts. We gave it publicity and it forced the Lagos State Government to begin paying attention to the issue [media professional, female, print]

### What researchers should know while using the media for evidence translation

To take advantage of the media for evidence translation of health systems research evidence, there are important considerations for the researchers. We found six of them, which are political arrangement of the media outfit, public perception, coverage, knack, content and relationship. The political affiliation and public perception of the media outfit can influence how the research findings are received. Research findings communicated through media outfits considered to be managed by an opposition political bloc could be received as propagandas, which could impact on how the researchers are perceived. Researchers must take steps to address the potentialities of mischaracterization of themselves and their findings, which can be because of the kind of media they use for dissemination.

[…] researchers must ensure they state disclaimers when using media outfits that may pass on a wrong message to the public. State that the research being reported has nothing to do with the political orientation of the media you are using and detail the processes you took. If it is on radio, allow for phone-ins so that people can call and verify the findings […] [media professional, male, freelance]

Moreover, the strength of the media coverage is important.

Depending on who you want to listen to you, should determine the assessment of coverage. If you want a national outlook, go for media with that national coverage, and if you want regional or a particular community to hear your findings, then you go for the media that has reach in those communities. You do not just jump on the media, one must take time to assess these things […] [media professional, male, radio]

Some media organizations have grown a reputation in particular subjects over the years, such that it has become their identity. It could be politics, health, culture, lifestyle, etc. The researchers should identify if their selected media for research communication has a knack for the findings they want to share. They can also explore the possibility of tailoring their research findings to fit the programme that has become the brand of the selected media.

[…] as researchers, you can learn how to leverage those programmes some media outfits have been known for in the public domain. Of course, it takes time to build a brand. So, it will be nice you organize your research to fit into the brand identity of the media you want to use. That way, the editor of the programme will easily accept you at a discounted rate or even for free, and you will have your research reaching out to many people [media professional, female, radio]

A media professional quickly added content, stating that the content of a research output for the media differs from that of academic publications.

Like in research, you start by writing the background, but in the media, we prioritize the results and write the background toward the end. Also, in recent times, contents with infographics travel a lot more than content with just plain texts. The paragraphs are usually shorter than what you see in academic publications, and you make more use of active descriptions that will elicit curiosity and facilitate understanding as well. For example, you do not say ‘a dog bit a boy and he died’. You rather say, ‘the boy died because he was bitten by a dog’. In the first, ‘he’ is not clear, as someone may still think of ‘he’ as the ’dog’ and not the boy [media professional, male, freelance]

**Fig. 1:**
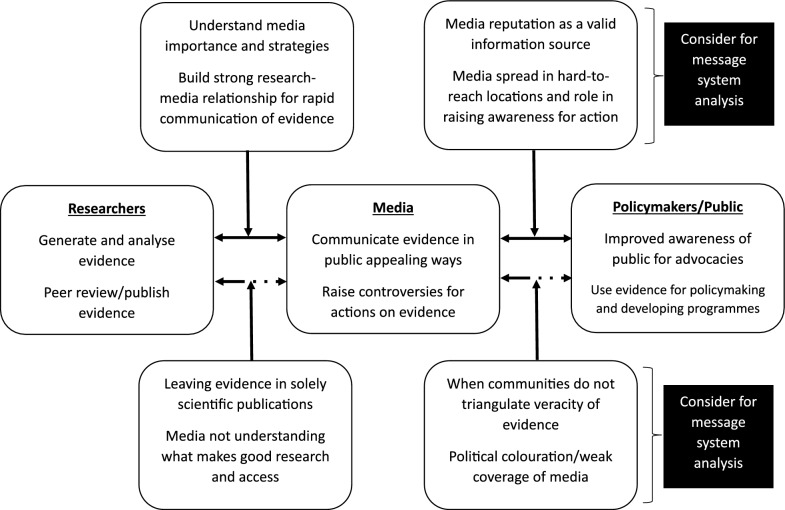
Research-media engagement framework for evidence translation of health systems and policy research. Alternate text: figure showing how the media sits in a mediating position between researchers in health systems and the public and policy-makers. The figure also shows concerns that the researchers should take into consideration for media analysis as they identify the media that should be used for evidence translation. Some of these considerations include the spread and political colouration of the media, helping the media to identify and understand credible research, researchers building relationships with the media, etc.

Finally, it was emphasized that researchers needed to foster relationships with the media for seamless transfer of research evidence from the researchers to the media, and then to the public and policy-makers. The media professionals strongly advised researchers to pay courtesy visits to some media outfits and regularly update the media with the evidence they have, including a directory of research experts and their respective fields for the media to contact when needed. Additionally, engagement with the media should not be an add-on, but an integral part of the overall research process. To pull together our findings, Fig. 1 provides a summary of research-media engagement for evidence translation.

## Discussion

Our findings show that the use of mass media in HPSR in Nigeria is an emerging area that could be harnessed and used to improve communication of research findings for policy making and community impact. We organized sessions for researchers and media experts to discuss how this can be encouraged, including the constraints that need to be addressed for effective translation of research evidence for policy actions using mass media. Notably, identifying and accessing quality studies and the complexities of scientific publications in terms of presentation and access, were issues identified by the media professionals as constraining the use of research evidence by the media and policy-makers.

For research to make sense to the media, language is key, hence researchers must learn and prioritize communication of research evidence using simple expressions in widely accessible platforms, such as blogs and news commentaries [35]. Media experts can help transfer these skills to researchers and justify the need for and impact of such skills by providing evidence of repeated reactions of policy-makers and the public to their outputs. There is evidence elsewhere of academics being trained to utilize the media for high research impact, which is measured by public awareness and understanding of their conducted research as well as policy response from government actors [14, 36]. Several studies have reported that academics lack soft skills, such as news-based reporting and social media use for research, hence they experience difficulties engaging policy-makers and the public [37, 38]. The context of evidence translation evolves, and if academics must make impacts with their research, they must ensure they evolve as well by learning the needed soft skills for facilitating communication and relationships with users of evidence [39].

An important finding in this study is the agreement between the researchers and media professionals that the typical academic means of communicating research evidence through academic journals is not doing enough and is restrictive to academic audiences. Even within academia, journal articles could still be restrictive to diverse disciplines and regions. Confirming this is a computation that showed that in about a year, a published blog had over 6200 reads, compared with an open access journal article with less than 1000 downloads in about 2 years [7]. This speaks to the concerns listed by the participants about the limitations of evidence documented in traditional academic outlets and using typical academic styles, which impede access and use, even by policy-makers.

The consensus among the researchers and media professionals who participated in the study is to utilize the comparative advantages of media over academia in terms of coverage and simplicity of presentation in enhancing evidence translation and use. Insights from the cultivation theory presented the importance of ‘two credibilities’ under institutional analysis, where media credibility is synergized with academia credibility for enhanced trust in and spread of research evidence for public awareness and policy impact [29].

Contingent on the above, public confidence and trust in the media is one comparative advantage the media enjoys, which is why the media is the most efficient means of reaching the grassroots. Political actors tend to react more swiftly to media reports than they will to documented evidence in academic papers, largely because of restrictions to access and understanding of academic papers owing to how technical and specialized they are [21].

Although there is paucity of studies on how political actors and policy-makers react to academic publications, but closely related studies are of the view that media attention to issues highly influences political agenda [40, 41]. This is unsurprising because of the reputation the media has in terms of rapid spread of information among the grassroots who are the obvious determinants of political legitimacy, especially in democratic contexts. In corroboration, the political-economy of the media and science communication has been a topical subject in climate change, with researchers encouraged to strategically hang on the media to get through to politicians who rely on the media for political legitimacy [42].

Gerbner affirmed the foregoing in explaining the cultivation theory, arguing that the media takes advantage of its ‘publicness’ to cultivate and influence public opinions towards informing awareness, advocacies, policy formulation and policy directions [23]. The significance of targeting the public for policy actions is manifested in recent works on health systems governance, recommending more grassroots-oriented approaches to influencing policies [43–46]. Undoubtedly, media involvement in research communication can help target grassroots with research evidence, in ways that will improve their awareness of issues and cause them to chase actions.

Our findings show that researchers expressed concerns about poor feedback to the communities where they source their data, which if addressed can contribute to stimulating awareness and demand for policy attention to the plights of communities. Media coverages are designed to reach nooks and crannies, and this can be leveraged for targeted communication to communities where the researchers conducted their studies. This means that the researchers will not be leveraging coverage alone, but trust and confidence often vested in the media by the public [8, 47].

To this end, researchers are advised to include media assessment in building research plans and dissemination strategies and ensuring that media usage is integral to the research and not an add-on. Besides assessing the media for trust and coverage when communicating research evidence, other important factors to consider include political affiliation and public perception of the media outfit, as well as the reputation of its contents in the public domain. These factors could influence how the public receives the evidence from the media and even how the researchers are perceived, which could survive over a long period. Interestingly, a part of the cultivation theory emphasizes message system analysis, which implies an analysis of public message contents and processes to understand uptake and utilization barriers and facilitators [29].

Moreover, as part of the relationship researchers should build with media professionals, forums for knowledge exchange are vital. Through such forums, issues about identifying, accessing and reporting the contents of quality research can be addressed. So, our findings recommend that frameworks on Getting Research into Policy and Practice (GRIPP) and evidence translation (for example, Uzochukwu et al. [21]) should be revised with the inclusion of the media as a cardinal bloc in HPSR. In fact, a systematic review of 159 knowledge translation theories in public health and health systems found that they were more commonly used to inform planning, implementation and evaluation of policies and programmes, as against dissemination, sustainability and scalability [48]. The research–media engagement framework (see Fig. 1) we have introduced in this study, contributes exceptionally to the health communication theoretical space in the direction of evidence dissemination for policy impact. Additionally, the cultivation theory provides theoretical analogy of cultivating the public and policy-making space for policy actions by using the messaging system, processes and effects of the media. The theory establishes that synergized impacts for evidence use in the policy context of health systems can be achieved by leveraging ‘two credibilities’ residing in the academia and media.

## Limitation

The current study is limited by the absence of policy-makers among the participants. Their presence could contribute to strengthening the evidence we have, by providing first-hand experiences of policy actions enabled differently by both the media and typical academic publications. We recommend future studies take note of this.

## Conclusions

Nevertheless, our study has shown the importance of media to evidence translation, and how researchers in HPSR can harness the strengths of the media in connecting with policy-makers and the public to stimulate conversations and actions of policy interest. Researchers in HPSR may begin to gravitate toward ‘commonalization’ of research evidence, which implies making sure that the public and policy-makers relate to research evidence just as they will do to media contents because of simplicity, accessibility, uniqueness to contexts and wider reach. They should equally foster collaborations with widely read blog and news outlets that present scientific information using journalistic skills. Cultivating public and policy-makers’ attention should become central to researchers in HPSR, and an integral part of the research process. Infusing media communication in HPSR communication should become the gold standard and a common practice in the bid to promote and strengthen evidence-informed policy-making in the health systems of Nigeria and across the globe.

## Data Availability

No datasets were generated or analysed during the current study.

## References

[1] Thoma B, Murray H, Huang SYM, Milne WK, Martin LJ, Bond CM, et al. The impact of social media promotion with infographics and podcasts on research dissemination and readership. Can J Emerg Med. 2018;20(2):300–6. 10.1017/cem.2017.394 28899440

[2] Huang S, Martin LJ, Yeh CH, Chin A, Murray H, Sanderson WB, et al. The effect of an infographic promotion on research dissemination and readership: a randomized controlled trial. Can J Emerg Med. 2018;20(6):826–33. 10.1017/cem.2018.436 30289098

[3] Wilson F, Umar MA. The effect of fake news on Nigeria’s democracy within the premise of freedom of expression. Glob Media J. 2019;17(32):1–12.

[4] Grossman E. Media and policy making in the digital age. Annu Rev Polit Sci. 2022;25:443–61.

[5] Roger M. Publishing research-based news articles: Opportunities and challenges for creating effective knowledge translation. Issues Educ Res. 2024;34(2):699–718.

[6] Bou-Karroum L, El-Jardali F, Hemadi N, Faraj Y, Ojha U, Shahrour M, et al. Using media to impact health policy-making: an integrative systematic review. Implement Sci. 2017;12(1):1–14. 28420401 10.1186/s13012-017-0581-0 PMC5395744

[7] Agwu P, Onwujekwe O. “Everyone!! wants to read a blog but your scholar-colleagues want to read your journal paper”: Research communication for wider reach 2021. https://hprgunn.com/everyone-wants-to-read-a-blog-but-your-scholar-colleagues-want-to-read-your-journal-paper/.

[8] Reed JE, Howe C, Doyle C, Bell D. Simple rules for evidence translation in complex systems: a qualitative study. BMC Med. 2018;16:1–20. 10.1186/s12916-018-1076-9 PMC6009041 29921274

[9] Schmitt T, Czabanowska K, Schröder-Bäck P. What is context in knowledge translation? Results of a systematic scoping review. Health Res Policy Syst. 2024;22(1):52. 38685073 10.1186/s12961-024-01143-5 PMC11057149

[10] Guggenheim L, Jang SM, Bae SY, Neuman WR. The dynamics of issue frame competition in traditional and social media. Ann Am Acad Pol Soc Sci. 2015;659(1):207–24.

[11] Chinn S, Hasell A. How different uses of social media inform perceptions of offline social norms and changes in vaccine intentions. Health Commun. 2023. 10.1080/10410236.2023.2207284. 37143302 10.1080/10410236.2023.2207284

[12] Niederdeppe J, Farrelly MC, Wenter D. Media advocacy, tobacco control policy change and teen smoking in Florida. Tob Control. 2007;16(1):47–52. 17297073 10.1136/tc.2005.015289 PMC2598450

[13] Oxman AD, Lewin S, Lavis JN, Fretheim A. SUPPORT Tools for evidence-informed health Policymaking (STP) 15: engaging the public in evidence-informed policy-making. Health Res Policy Syst. 2009;7(1):S15. 20018105 10.1186/1478-4505-7-S1-S15 PMC3271826

[14] Stewart EC, Davis JS, Walters TS, Chen Z, Miller ST, Duke JM, et al. Development of strategies for community engaged research dissemination by basic scientists: a case study. Transl Res. 2023;252:91–8. 36108910 10.1016/j.trsl.2022.09.001

[15] Petkovic J, Duench S, Trawin J, Dewidar O, Pardo JP, Simeon R, et al. Behavioural interventions delivered through interactive social media for health behaviour change, health outcomes, and health equity in the adult population. Cochrane Database Syst Rev. 2021. 10.1002/14651858.CD012932.pub2. 34057201 10.1002/14651858.CD012932.pub2 PMC8406980

[16] Chen M, Yu W, Cao X. Experience pandemic fatigue? Social media use may play a role: testing a model of pandemic fatigue development from a social media perspective. Health Commun. 2022;38(14):1–11. https://www.tandfonline.com/doi/full/10.1080/10410236.2022.2149095. 10.1080/10410236.2022.2149095 36419354

[17] Adegboyega A. Buhari approves 159 new radio, television stations. Premium Times. 2021. https://www.premiumtimesng.com/news/top-news/487197-buhariapproves-159-new-radio-television-stations.html?tztc=1.

[18] Statista. Share of households owning a television in Nigeria as of 2020, by area 2022. https://www.statista.com/statistics/1268960/households-with-atelevision-at-home-in-nigeria-by-area/.

[19] Njoku G. Continued relevance of radio in digital age. The Guardian. 2022. https://guardian.ng/art/continued-relevance-of-radio-in-digital-age/.

[20] DataReportal. Digital 2023: Nigeria 2023. https://datareportal.com/reports/digital-2023-global-overview-report.

[21] Uzochukwu B, Onwujekwe O, Mbachu C, Okwuosa C, Etiaba E, Nyström ME, et al. The challenge of bridging the gap between researchers and policy makers: experiences of a Health Policy Research Group in engaging policy makers to support evidence informed policy making in Nigeria. Glob Health. 2016;12(1):1–15. 10.1186/s12992-016-0209-1 PMC5095957 27809862

[22] Ezenwaka U, Mbachu C, Etiaba E, Uzochukwu B, Onwujekwe O. Integrating evidence from research into decision-making for controlling endemic tropical diseases in South East Nigeria: perceptions of producers and users of evidence on barriers and solutions. Health Res Policy Syst. 2020;18:1–10. 31931821 10.1186/s12961-019-0518-y PMC6958705

[23] Gerbner G. An institutional approach to mass communications research. Communication theory and research: proceedings of the first international symposium. Springfield: Charles C. Thomas; 1967.

[24] Gerbner G. Toward" cultural indicators": the analysis of mass mediated public message systems. AV Commun Rev. 1969. 10.1007/BF02769102.

[25] Tian Y, Yoo JH. Medical drama viewing and medical trust: a moderated mediation approach. Health Commun. 2020;35(1):46–55. 30358426 10.1080/10410236.2018.1536959

[26] Gerbner G. Cultural indicators: the case of violence in television drama. Ann Am Acad Pol Soc Sci. 1970;388(1):69–81.

[27] Dutta MJ, Kaur-Gill S, Tan N. Cultivation in health and risk messaging. In: Dutta MJ, Kaur-Gill S, Tan N, editors. Oxford research encyclopedia of communication. Oxford: Oxford University Press; 2017.

[28] Chung JE. Medical dramas and viewer perception of health: testing cultivation effects. Hum Commun Res. 2014;40(3):333–49.

[29] Potter WJ. A critical analysis of cultivation theory. J Commun. 2014;64(6):1015–36.

[30] Rodriguez A, Smith J. Phenomenology as a healthcare research method. Royal Coll Nurs. 2018. 10.1136/eb-2018-102990. 10.1136/eb-2018-102990 30201830

[31] Dunne C. The place of the literature review in grounded theory research. Int J Soc Res Methodol. 2011;14(2):111–24.

[32] Tong A, Sainsbury P, Craig J. Consolidated criteria for reporting qualitative research (COREQ): a 32-item checklist for interviews and focus groups. Int J Qual Health Care. 2007;19(6):349–57. 17872937 10.1093/intqhc/mzm042

[33] Neubauer B, Witkop C, Varpio L. How phenomenology can help us learn from the experiences of others. Perspect Med Educ. 2019;8:90–7. 30953335 10.1007/s40037-019-0509-2 PMC6468135

[34] Neubauer B, Witkop C, Varpio L. How phenomenology can help us learn from the experiences of others. Perspect Med Educ. 2019;8(2):90–7. 30953335 10.1007/s40037-019-0509-2 PMC6468135

[35] Mbachu C, Agwu P, Obi F, Onwujekwe O. Understanding and bridging gaps in the use of evidence from modeling for evidence-based policy making in Nigeria’s health system. MDM Policy Pract. 2024;9(1):23814683231225656. 38250666 10.1177/23814683231225658 PMC10798080

[36] Jordan K. Academics’ perceptions of research impact and engagement through interactions on social media platforms. Learn Media Technol. 2022. 10.1080/17439884.2022.2065298.

[37] Kalbarczyk A, Rodriguez DC, Mahendradhata Y, Sarker M, Seme A, Majumdar P, et al. Barriers and facilitators to knowledge translation activities within academic institutions in low-and middle-income countries. Health Policy Plan. 2021;36(5):728–39. 33661285 10.1093/heapol/czaa188 PMC8173595

[38] Chan TM, Dzara K, Dimeo SP, Bhalerao A, Maggio LA. Social media in knowledge translation and education for physicians and trainees: a scoping review. Perspect Med Educ. 2020;9:20–30. 31834598 10.1007/s40037-019-00542-7 PMC7012997

[39] Borst RA, Wehrens R, Nsangou M, Arikpo D, Esu E, Al Metleq A, et al. What makes knowledge translation work in practice? Lessons from a demand-driven and locally led project in Cameroon, Jordan and Nigeria. Health Res Policy Syst. 2023;21(1):127. 38049826 10.1186/s12961-023-01083-6 PMC10694879

[40] Helfer L. Media effects on politicians: an individual-level political agenda-setting experiment. Int J Press/Politics. 2016;21(2):233–52.

[41] Helfer L, Van Aelst P. Why politicians react to media coverage: a comparative experiment<? br?> of political agenda-setting. Agenda Setting J. 2020;4(1):88–108.

[42] Meribe NC. The political economy of climate change reporting in Nigeria. Afr J Stud. 2017;38(1):40–65.

[43] Abimbola S. The uses of knowledge in global health. BMJ Spec J. 2021. 10.1136/bmjgh-2021-005802. 10.1136/bmjgh-2021-005802 PMC8030475 33820807

[44] Khan MH. Political settlements and the analysis of institutions. Afr Aff. 2018;117(469):636–55.

[45] Odii A, Onwujekwe O, Hutchinson E, Agwu P, Orjiakor CT, Ogbozor P, et al. Absenteeism in primary health centres in Nigeria: leveraging power, politics and kinship. BMJ Glob Health. 2022;7(12): e010542. 36593645 10.1136/bmjgh-2022-010542 PMC9730370

[46] Reimer-Kirkham S, Jule A. Crosstalk: public cafes as places for knowledge translation concerning health care research. Health Commun. 2015;30(5):496–503. 24992638 10.1080/10410236.2013.868398

[47] Hamid AM, Tamam E, Bin Osman MN. Relationships between media exposure and knowledge, attitude, and practice on HIV/AIDS: a cross sectional survey of adolescent Islamiyya girls in Nigeria. Health Commun. 2019. 10.1080/10410236.2018.1564960. 10.1080/10410236.2018.1564960 30700145

[48] Strifler L, Cardoso R, McGowan J, Cogo E, Nincic V, Khan PA, et al. Scoping review identifies significant number of knowledge translation theories, models, and frameworks with limited use. J Clin Epidemiol. 2018;100:92–102. 29660481 10.1016/j.jclinepi.2018.04.008

